# Clinical and molecular outcomes from the 5-Year natural history study of SSADH Deficiency, a model metabolic neurodevelopmental disorder

**DOI:** 10.1186/s11689-024-09538-9

**Published:** 2024-04-24

**Authors:** Itay Tokatly Latzer, Jean-Baptiste Roullet, Wardiya Afshar-Saber, Henry H. C. Lee, Mariarita Bertoldi, Gabrielle E. McGinty, Melissa L. DiBacco, Erland Arning, Melissa Tsuboyama, Alexander Rotenberg, Thomas Opladen, Kathrin Jeltsch, Àngels García-Cazorla, Natalia Juliá-Palacios, K. Michael Gibson, Mustafa Sahin, Phillip L. Pearl

**Affiliations:** 1 Department of Neurology, Boston Children’s Hospital, Harvard Medical School, 300 Longwood Ave, Boston, MA 02115 USA; 2 School of Medicine, Faculty of Medical and Health Sciences, Tel-Aviv University, Tel Aviv, Israel; 3 Department of Pharmacotherapy, College of Pharmacy and Pharmaceutical Sciences, Washington State University, Spokane, WA USA; 4 Rosamund Stone Zander Translational Neuroscience Center, Boston Children’s Hospital, Boston, MA 02115 USA; 5 F.M. Kirby Neurobiology Center, Boston Children’s Hospital, Boston, MA 02115 USA; 6 Department of Neuroscience, Biomedicine and Movement Sciences, University of Verona, Verona, Italy; 7 Institute of Metabolic Disease, Baylor Scott & White Research Institute, Dallas, TX USA; 8 Division of Neuropediatrics & Metabolic Medicine, University Children’s Hospital Heidelberg, Im Neuenheimer Feld 430, 69120 Heidelberg, Germany; 9 Neurometabolic Unit, Neurology Department, Institut de Recerca, Hospital Sant Joan de Déu, Barcelona, Spain

**Keywords:** Succinic semialdehyde dehydrogenase, Neurotransmitters, GABA, Development

## Abstract

**Background:**

Succinic semialdehyde dehydrogenase deficiency (SSADHD) represents a model neurometabolic disease at the fulcrum of translational research within the Boston Children’s Hospital Intellectual and Developmental Disabilities Research Centers (IDDRC), including the NIH-sponsored natural history study of clinical, neurophysiological, neuroimaging, and molecular markers, patient-derived induced pluripotent stem cells (iPSC) characterization, and development of a murine model for tightly regulated, cell-specific gene therapy.

**Methods:**

SSADHD subjects underwent clinical evaluations, neuropsychological assessments, biochemical quantification of γ-aminobutyrate (GABA) and related metabolites, electroencephalography (standard and high density), magnetoencephalography, transcranial magnetic stimulation, magnetic resonance imaging and spectroscopy, and genetic tests. This was parallel to laboratory molecular investigations of in vitro GABAergic neurons derived from induced human pluripotent stem cells (hiPSCs) of SSADHD subjects and biochemical analyses performed on a versatile murine model that uses an inducible and reversible rescue strategy allowing on-demand and cell-specific gene therapy.

**Results:**

The 62 SSADHD subjects [53% females, median (IQR) age of 9.6 (5.4–14.5) years] included in the study had a reported symptom onset at ∼ 6 months and were diagnosed at a median age of 4 years. Language developmental delays were more prominent than motor. Autism, epilepsy, movement disorders, sleep disturbances, and various psychiatric behaviors constituted the core of the disorder’s clinical phenotype. Lower clinical severity scores, indicating worst severity, coincided with older age (*R*= -0.302, *p* = 0.03), as well as age-adjusted lower values of plasma γ-aminobutyrate (GABA) (*R* = 0.337, *p* = 0.02) and γ-hydroxybutyrate (GHB) (*R* = 0.360, *p* = 0.05). While epilepsy and psychiatric behaviors increase in severity with age, communication abilities and motor function tend to improve. iPSCs, which were differentiated into GABAergic neurons, represent the first in vitro neuronal model of SSADHD and express the neuronal marker microtubule-associated protein 2 (MAP2), as well as GABA. GABA-metabolism in induced GABAergic neurons could be reversed using CRISPR correction of the pathogenic variants or mRNA transfection and SSADHD iPSCs were associated with excessive glutamatergic activity and related synaptic excitation.

**Conclusions:**

Findings from the SSADHD Natural History Study converge with iPSC and animal model work focused on a common disorder within our IDDRC, deepening our knowledge of the pathophysiology and longitudinal clinical course of a complex neurodevelopmental disorder. This further enables the identification of biomarkers and changes throughout development that will be essential for upcoming targeted trials of enzyme replacement and gene therapy.

## Introduction

**Fig. 1:**
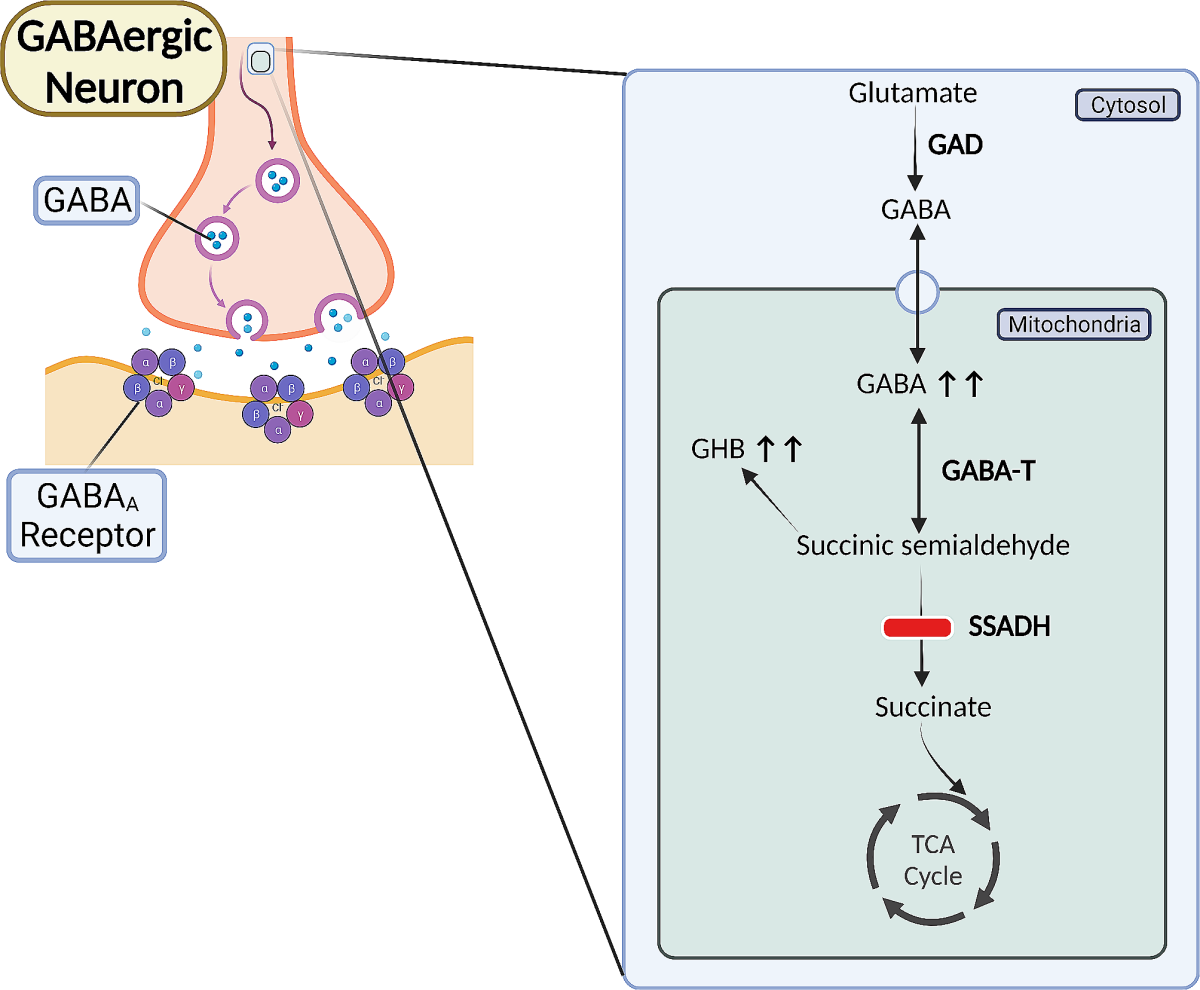
The metabolic consequences of an impaired or lacking SSADH enzyme include the accumulation of the inhibitory neurotransmitters GABA and GHB. GABA- γ-aminobutyrate; GABA-T- GABA Transaminase; GAD- Glutamic Acid Decarboxylase; GHB- γ-hydroxybutyrate; SSADH- Succinic Semialdehyde Dehydrogenase; TCA- Tricarboxylic Acid Cycle

Succinic semialdehyde dehydrogenase deficiency (SSADHD) (OMIM #271,980) is an autosomal recessive inherited metabolic disorder of γ-aminobutyric acid (GABA) catabolism with an estimated pan-ethnic prevalence of ∼ 1/460,000 [1]. It was first postulated that SSADH deficiency leads to increased excretion of γ-hydroxybutyrate (GHB) and the associated neurological and developmental abnormalities in 1981 [2]. During the following decades, multiple case reports and laboratory-based studies established the evidence that the primary metabolic defect of SSADHD involves the GABA degradation pathway [3–13]. Deficiency or absence of SSADH, a mitochondrial enzyme, ensues in the accumulation of GABA and other metabolites such as GHB (Fig. 1). The genetic basis of SSADHD was first reported in 1998 [14], and since then, a number of case series and cohort studies have described the clinical and biochemical phenotypes of SSADHD resulting from bi-allelic pathogenic variants in *ALDH5A1* [15–20]. Since different combinations of *ALDH5A1* variants may ensue in variable structural and functional impairments of the resultant SSADH enzyme, the clinical presentation of SSADHD is broad in the degree of severity [21, 22]. Considering the dominant presence of GABA in the basal ganglia, neuropathological findings of striking discoloration of the globus pallidi [23] and neuroimaging findings of (MRI)-derived T2-weighted hyperintensity in the globus pallidi and subthalamic nuclei, along with magnetic resonance spectroscopy (MRS)-derived increased GABA/N-acetylaspartate (NAA) ratios [24], suggest this is one of the primary areas involved in the clinical manifestations of the disorder. Moreover, compared to healthy controls, individuals with SSADHD have been shown to have an increased burden of enlarged perivascular spaces in several areas including the basal ganglia [25]. The main manifestations of SSADHD are cognitive, adaptive, behavioral, communication, and language deficits, epilepsy, movement disorders, and psychiatric traits such as anxiety, hyperactivity, and obsessive-compulsiveness. The clinical onset of SSADHD typically occurs during the first year of life with developmental delays. Developmental course and outcome always seem to be affected, ranging from individuals who have a mild intellectual disability, borderline low adaptive functions, and are completely ambulatory to those with a profound intellectual disability, complete dependency in terms of daily living, and are non-ambulatory [26]. The diagnosis of SSADHD typically follows a differential diagnosis-based biochemical and genetic investigation stemming from the nonspecific phenotype. Ongoing efforts aim to establish enzyme and gene replacement therapies for SSADHD [27]; however, treatments are currently symptomatic and supportive.

The SSADHD Natural History Study (NHS) (ClinicalTrials.gov ID: NCT03758521) is a multinational prospective study established in 2019 at Boston Children’s Hospital (BCH) by investigators of the SSADH Deficiency Research Consortium (NIH R01 1R01HD091142) [28]. The study’s leading clinical site is BCH, and it includes two more international clinical sites: University Children’s Hospital Heidelberg (UCHH) in Germany and Hospital Sant Joan Déu Barcelona Children’s Hospital (UDB) in Spain. The University of Florida provides the study’s data core, and *in silico* and bioanalytical variant analyses are completed at the University of Verona, Italy. The study’s primary objectives are establishing and validating a formal clinical severity score for SSADHD and identifying biomarkers predicting clinical severity. This was achieved by correlating clinical outcomes including neuropsychologic assessments with genetic, biochemical, neurophysiological, and neuroimaging measures. The clinical battery of tests that individuals with SSADHD undergo include the standard existing clinical and neuropsychological measures reflecting the known clinical manifestations. Notably, the 5-year duration of this study has only begun to allow assessment of more longitudinal outcomes of its participants. Translational directions stemming from the study included the investigation of the molecular neurobiological pathomechanisms of SSADHD as part of developing genetic-based therapies for this disorder. This consisted of developing an in vitro model of SSADHD from human induced pluripotent stem cells (hiPSC) differentiated into GABAergic neurons and through biochemical analyses performed on our novel *ALDH5A1*^*lox − STOP*^ mouse.

## Methods

### Settings and population

The SSADHD NHS is approved by the Boston Children’s Hospital Institutional Review Board (#P00029917). SSADHD subjects are recruited through the SSADHD Association and the International Working Group on Neurotransmitter Related Disorders (iNTD) (http://intd-online.org/). Genetically confirmed SSADHD subjects are included. Once enrolled, study participants undergo a biennial in-person clinical assessment, including a neurological examination, neuropsychological evaluation, neuroimaging consisting of magnetic resonance imaging (MRI) and magnetic resonance spectroscopy (MRS), electroencephalography (EEG), blood collection for GABA, GHB, and γ-guanidinobutyrate (GBA), magnetoencephalography (MEG), transcranial magnetic stimulation (TMS) studies, and collection of skin fibroblasts used for the differentiation of hiPSCs into GABAergic neurons, constituting the basis of the SSADHD in vitro model. Notably, not all study participants are able to complete the full set of the assessments mentioned above, as not all participating sites have the required staffing and facilities (for example, biopsies deriving human iPSCs are only done at the BCH site owing to its available laboratory). Additionally, some participants have a low tolerance to lengthy procedures done without sedation (such as electroencephalography or neuroimaging), tests requiring sustained cooperation (such as transcranial magnet stimulation), or assessments necessitating certain motor abilities (such as ADOS-2). Some participants could not tolerate the neuroimaging, neurophysiologic, and neuropsychiatric assessments. This limitation is common in rare disease research, especially when affected individuals have neuropsychiatric impairment. An additional “standard of care” group of SSADHD individuals unable to attend the on-site evaluations in person sends their relevant medical records to one of the clinical sites and has laboratory tests taken at home and then shipped to the study’s laboratory sites.

### Clinical severity assessment

Following their medical and neurological examination, participants’ clinical severity is assessed bedside by means of a validated SSADHD-specific clinical severity score (CSS) [21]. The CSS, ranging from 5 (most severe) to 25 (least severe), is formulated by the compounded severity scores of five domains representing the primary manifestations of the disorder: cognitive, communication, motor, psychiatric, and epilepsy (each domain is scored on a 1–5 scale, 1 indicating the most severity). The CSS was developed by the primary investigators of the SSADHD NHS, based on their longstanding clinical experience and data collected from an ongoing Boston Children’s Hospital-centered SSADHD registry in which SSADHD subjects (or their legal caregivers) complete twice yearly an electronic survey including an elaborate collection of questions about their clinical characteristics [29]. The CSS is practical and applicable to every clinical setting, as it is administered bedside. Its recent validation has been demonstrated by the significant correlation of its subscales with their respective standardized objective metrics. Additional CSS-related analyses showed that the general clinical severity, and specifically that of the epilepsy and psychiatric manifestations of individuals with SSADHD, increases with age but communication skills may improve with age [21].

### Neuropsychological, behavioral, and sleep-related assessments

Study participants undergo a comprehensive neuropsychological evaluation assessing cognitive abilities [Differential Abilities Scale (DAS), 2nd Edition [30], Wechsler Abbreviated Scales of Intelligence (WASI) [31], or Mullen Scales of Early Learning (MSEL) [32] were administered in a standardized manner. Children 2.6-17.11 years old were first administered the DAS-II (*N* = 13), older individuals were first administered the WASI (*N* = 8), and for participants who were not able to complete the basal items on these measures, the clinician changed to the MSEL, which is designed for individuals with skills under 4 years of age (*N* = 11). Standard scores or ratio IQ scores were used for analysis.]; adaptive functions (Vineland Adaptive Behavior Scales, 2nd Edition [33]), receptive and expressive language skills [Receptive Language and Expressive Language, 3rd Edition (REEL-3) [34]], primary communication abilities [Autism Diagnostic Observation Scale-2 (ADOS-2) [35]], motor function [Movement Assessment Battery for Children- 2nd Edition (MABC-2) [36]], and behavioral and psychiatric traits [Achenbach Child or Adult Behavior Checklist (CBCL or ABCL, respectively) [37–39]]. To assess sleep disturbances such as bedtime resistance, sleep onset delay, sleep duration, sleep anxiety, daytime sleepiness, night waking, parasomnias, and sleep-disordered breathing, participants’ caregivers fill out the *Children’s Sleep Habits Questionnaire (CSHQ)* [40].

### Biochemical metrics

Hydrolysis with 6 N HCl by stable isotope dilution liquid chromatography-mass spectrometry determines plasma GABA, GHB, and GBA concentrations [41].

### Neurophysiological assessments

Electroencephalography (EEG) recordings are obtained as 30-minute 21-channel digital studies (Natus® NeuroWorks® EEG Software, Natus Medical Incorporated, Ontario, Canada) of the awake and asleep states, with electrodes placed according to the 10/20 International System. Bipolar and referential electrode montage parameters included a 512 Hz sampling rate and 24-bit analog-to-digital conversion. A subset of patients underwent high-density EEG using a 64-channel system (eego^TM^mylab, ANT Neuro, Netherlands), with a 1 K Hz sampling rate and collecting somatosensory evoked responses using a puff of air to the fingers.

Transcranial magnetic stimulation (TMS) is administered through the Nexstim 5.1.1 system (Nexstim, Finland). Anatomical T1-weighted MRI sequences of study participants are used for co-registration and frameless stereotaxy. A figure-of-eight coil detects the primary motor cortex, and the contralateral abductor pollicis brevis muscle detects motor-evoked potentials [42]. Electromyography (EMG) is recorded at 3 kHz using a bandpass filter (10–500 Hz). TMS-derived outcomes include the resting motor threshold (rMT) [the minimum machine output (MO) required to induce a motor-evoked potential (MEP) above 50 µV from the target resting muscle in more than 50% of trials], cortical silent period (CSP) (the duration from stimulation at 150% rMT to the spontaneous return of voluntary muscle activity detected via EMG), and long-interval cortical inhibition (LICI) (the log transformation of the peak-to-peak amplitude of the second to the first pulse’s resultant MEPs, determined by pairs of stimulations delivered at 120% rMT with 100-milisecond interpulse intervals). LabChart v8.1.17 analyzes the EMG signals and extracts the CSP and LICI measurements.

### Neuroimaging

MRIs are completed with a whole-body 3-Tesla MRI scanner (Siemens Skyra, Erlangen, Germany). GABA/N-acetyl aspartate (NAA) ratio estimations derive from two acquisitions (TR 1500ms; TE 68ms; bandwidth 1200 Hz) of GABA-specific MEGA-PRESS sequences of one voxel (27cm^3^; 30 mm×30 mm×30 mm). The initial acquisition includes a 1.9 ppm-arranged editing pulse, enabling selective refocusing of the 3.0 ppm GABA multiplet. The sequential acquisition uses another location to allocate the inversion, determining the J-evolution of GABA. Considering MRI abnormalities of SSADHD individuals are most consistently identified in the basal ganglia, these areas are primarily sampled [24]. A sampling of the posterior cingulate and occipital cortices was added since these areas were proven reliable in demonstrating GABA measurements [43–45]. Spectroscopy data is processed by the LCModel9 software (version 6.3) [46].

### Genetic tests

GABA-related gene (*ALDH5A1, Abat, Glud1, GLS, Glul*) expression (normalized to *GAPDH* expression and expressed as 2^−ΔCt^) in whole blood was done by RNA extraction using a PAXgene Blood miRNA Kit (QIAGEN, cat no. 763,134, Hilden, Germany). RNA quality and concentration were achieved using a Fragment Analyzer System Kit (cat. no. DNF-472-0500, Agilent, Santa Clara, CA) and the Qubit RNA HS assay kit (Invitrogen, cat. no. Q32855, Waltham, MA). the RT2 First Strand Kit (QIAGEN, cat no. 330,404) and loaded into a 384-well custom RT2 Profiler array (QIAGEN, Hilden, Germany) was used to obtain cDNA. qPCR was completed through a CFX 384 (Bio-Rad Laboratories, Hercules, CA).

Structural *in-silico* analyses of the specific variant-derived types of substitution, residue localization, microenvironment, and interactions of SSADH (PDB: 2W8N (PDB: 2W8N [47]) were achieved by PyMOL Molecular Graphics System (version 2.5.2, Schrödinger LLC.). The web tool Mol* Viewer [48] was used to determine the molecular interactions between residues in a surrounding area of 5 Å.

### GHB analysis in mice

All animal housing and breeding procedures performed in this study were covered by protocols approved by the Institutional Animal Care and Use Committee at Boston Children’s Hospital and in accordance with the National Institutes of Health (NIH) Guide for the Care and Use of Laboratory Animals. Mice were housed in standard cages in a temperature-controlled facility with a 12-hour light/dark cycle and a continuous supply of water and food ad libitum. Both male and female mice were used in this study and the data presented. To construct this novel inducible *aldh5a1*^*lox − STOP*^ mice, we used a one-step mouse genome editing CRISPR/Cas9-mediated strategy [49], inserting a lox-STOP cassette disrupting the endogenous *aldh5a1* gene. The absence of endogenous SSADH expression was confirmed by western blot analysis, ensuring splicing integrity leading to obligated *aldh5a1* disruption [50, 51]. Survival of this novel *aldh5a1*^*lox − STOP*^ mouse has been characterized, exhibiting obligatory premature lethality around three weeks of postnatal age, phenocopying total *aldh5a1* knock-out mice [18]. Mice were sacrificed on postnatal day 16, using acute isoflurane anesthesia. Whole blood was collected and spotted directly on blood spot cards (903 Protein Saver Card, EBF #10,550,021), dried overnight, and stored at 4^o^C under desiccation. Extraction and quantitation of GHB from dry blood spots were carried out as described previously [52]. We collected whole blood from wild-type (WT), heterozygous (HET), and homozygous (HOM) mutant littermates at postnatal age P16. GHB content from *aldh5a1*^*lox − STOP*^ mice blood was determined from an established dry blood spot methodology [52].

### SSADHD-derived human induced pluripotent stem cells (hiPSC)

**Fig. 2:**
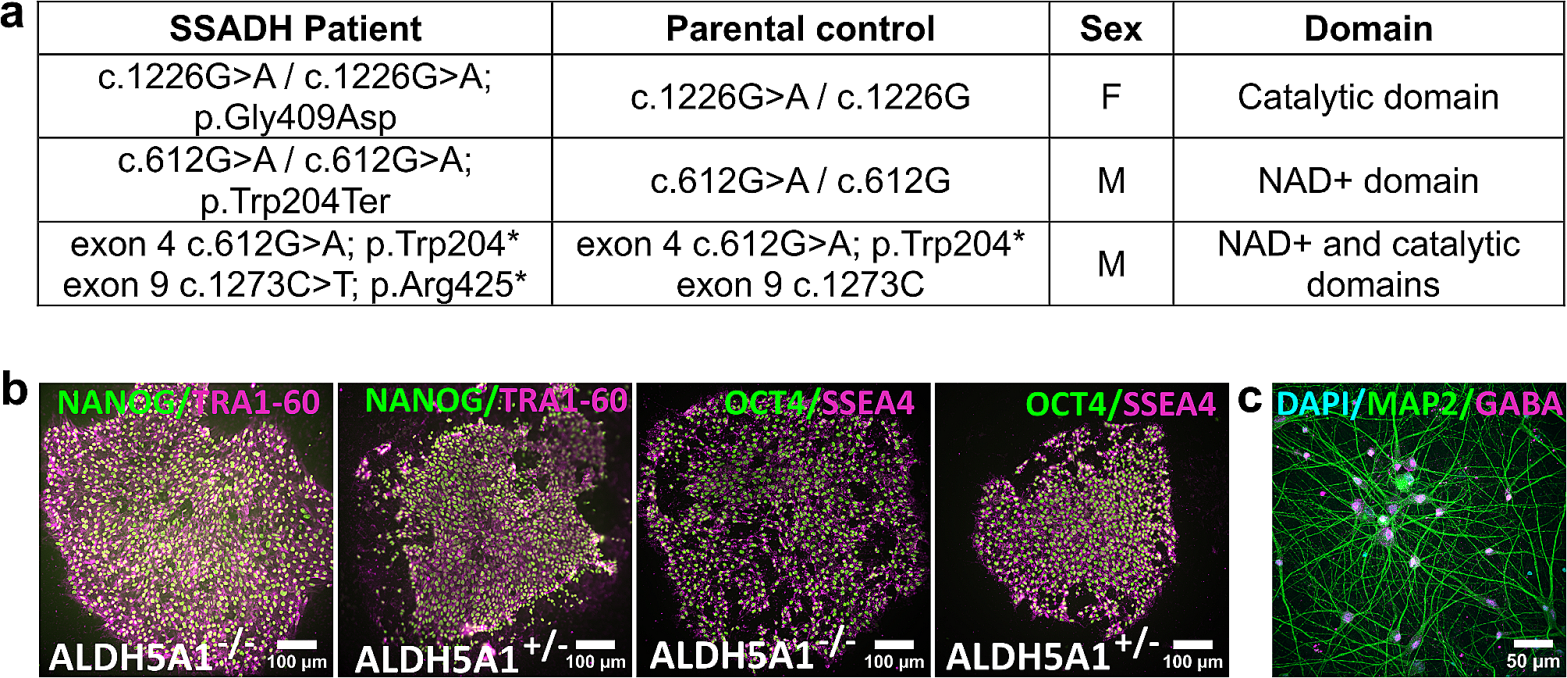
Summary and characterization of the human iPSCs lines from SSADH deficient patients and sex-matched unaffected parental controls. (**a**) Recapitulative table. (**b**) Representative images of the iPSCs (ALDH5A1-/- and ALDH5A1+/-) demonstrating the successful reprogramming by characterizing the expression of pluripotency markers NANOG (Nanog homeobox x in green) and TRA-1-60 (podocalyxin in magenta), OCT4 (octamer binding transcription factor 4 in green), and SOX2 (SRY-Box Transcription Factor 2 in magenta). (**c**) Representative image of iPSC-derived iGABA neurons labelled with DAPI (cyan), MAP2 (green), GABA (magenta). Scale bar 50 μm

SSADHD has been studied in vitro using traditional cell culture techniques and models such as HEK293 cells or patient fibroblasts [53, 54]. Although these systems offer advantages related to the cost of maintenance and ease of use, overexpression in HEK 293 may result in aberrant behavior of the protein due to aggregation, and these non-neuronal models may not recapitulate the SSADHD phenotypes. Despite the limitation of the fibroblasts model, the latter can be reprogrammed to induced pluripotent stem cells (iPSCs) with the capability, when cultured, of unlimited self–renewal and reproduction of all adult cell types in the course of their differentiation [55]. In this study, fibroblasts were obtained by standard punch biopsy from three SSADHD patients with mutations in the catalytic domain (c.1226G > A / c.1226G > A; p.Gly409Asp), in the NAD + binding domain (c.612G > A / c.612G > A; p.Trp204*) and NAD + binding domain/catalytic domain (exon 4 c.612G > A; p.Trp204* and exon 9 c.1273 C > T; p.Arg425*) and their sex-matched parental control (Fig. 2a). There were no developmental considerations when using parents as controls since the parents are obligate heterozygotes and asymptomatic. Fibroblasts were then reprogrammed into iPSCs at the Harvard Stem Cell Institute using a non-integrating Sendai virus to overexpress OCT4, SOX2, KLF4, and hc-MYC [55]. iPSCs were characterized following standard protocols [56], including karyotyping and pluripotency marker expression (Fig. 2b).

Additionally, modern genome editing methods, such as CRISPR-Cas9-based gene editing, can be used to introduce the mutation into control iPSCs or to create isogenic controls to correct SSADH-related mutation in patient-derived cells to distinguish biologically relevant cellular phenotypes that contribute to SSADH-related traits versus those resulting from individual variability between patients’ genetic backgrounds [57]. This approach was taken to generate neural stem cells to model SSADHD, showing increased GABA levels in ALDH5A1^−/−^. However, no other biochemical hallmarks of SSADHD were detected [58]. To overcome this limitation, iPSCs can be further differentiated into neurons in vitro to model SSADHD. This can be achieved using growth factor signaling to mimic developmental cues or, more recently, inducing neurons directly from iPSCs by overexpressing neurogenic transcription factors for cell fate conversion into various subtypes of neurons such as GABAergic or excitatory neurons [59, 60]. We differentiated the iPSCs into GABAergic neurons using induced DLX2 and ASCL1 (pLV-TetO-hDLX2-P2A-hASCL1-T2A-Puro) [56]. The ability to use specific transcription factors to induce the development of populations of highly enriched neurons for specific sub-types allows for drug screening and molecular and biochemical dissection of pathological processes in vitro that would be far more difficult in vivo.

### Statistical analyses

Data were analyzed using SPSS Statistics (IBM SPSS Statistics, Version 28, 2021, IBM Corp, Armonk, NY, USA). Categorical variables are reported as their relative frequencies, and continuous variables as either mean ± standard deviations (mean ± SD) or median and interquartile ranges (IQR), depending on their normality distribution. Age-adjusted patrial correlations were performed between biomarkers (gene expression, biochemical, neurophysiologic, and neuroimaging) and the CSS. For the analyses of GHB in mice, the amount of GHB in each sample was normalized by its internal standard and then calculated in µM concentration. All samples were compared to WT control levels. All data were included and statistically tested using One-way ANOVA followed by Dunnett’s multiple comparisons test in GraphPad Prism, represented as mean ± standard error of the mean (SEM). A *p* value ≤ 0.05 was considered significant for all analyses.

## Results

**Fig. 3:**
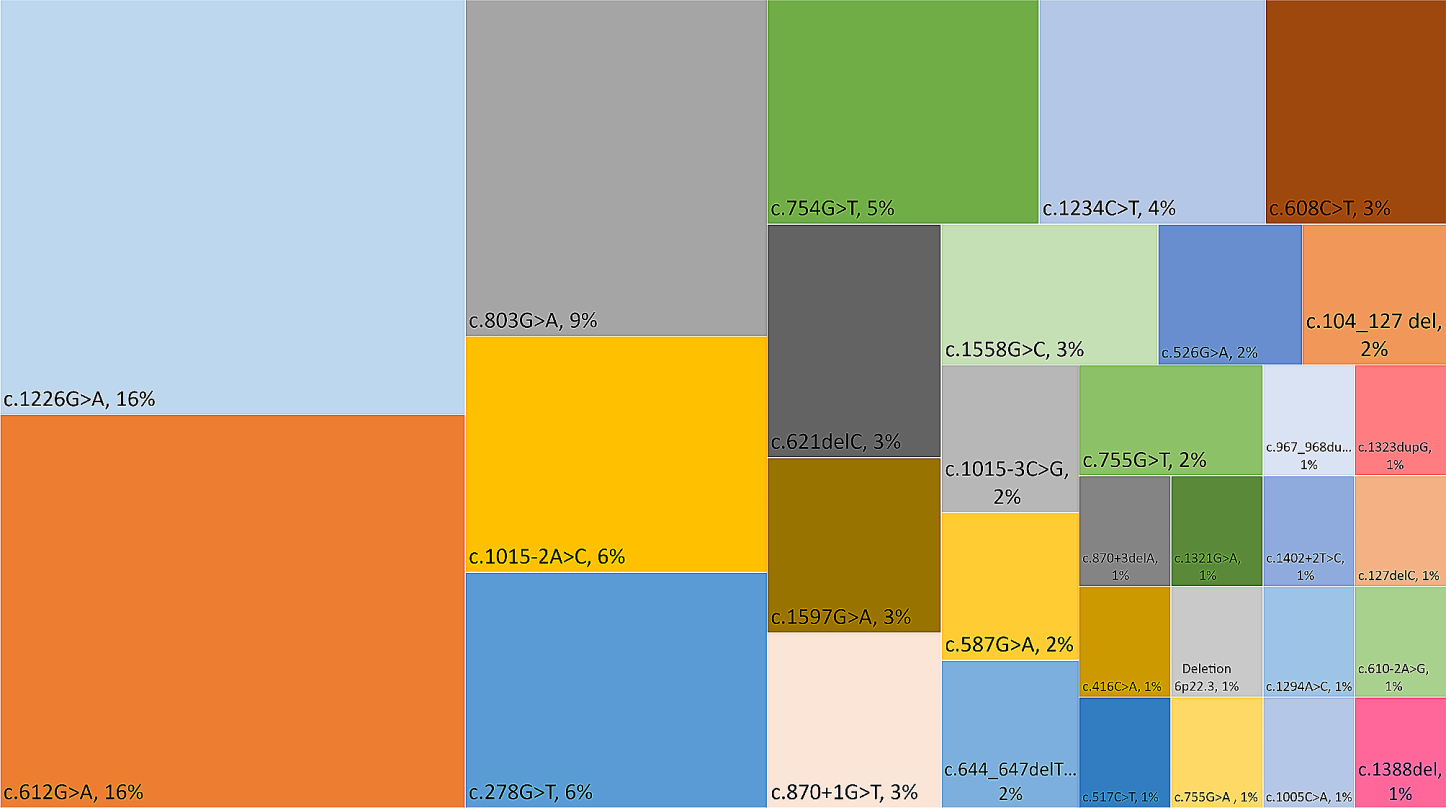
A treemap chart illustrating the occurrence rate of *ALDH5A1* variants in subjects enrolled in the SSADHD natural history study

At its 5-year mark, the SSADHD NHS includes 62 enrollees (53% females) with a median (IQR) age of 9.6 (5.4–14.5) years (full range 1.2–39.6 years), 29 (47%) of whom were recruited at BCH, 14 (23%) at UCHH, 10 (16%) at UDB, and 9 (14%) who were part of the SOC group. Thirteen (21%) of the study’s subjects were born to known consanguineous parents. 40% of the study group were homozygotes and 60% compound heterozygotes of the 32 pathogenic *ALDH5A1* variants, the most common of which were c.1226G > A (16%), c.612G > A (16%), and c.803G > A (9%). The complete list of ALDH5A1 variants along with their occurrence rate is displayed in Fig. 3. 55% of these variants led to missense mutations, 20% non-sense mutations, 14% splice-site errors, and 10% frameshift mutations (9% deletions and 1% duplications). An additional variant consisted of chromosome 6p22.3 deletion of 526 base-pairs, or 0.526 kB [arr(GRCh37) 6p22.3 (24528034_24528560)]. Part of the data provided has been described in the halfway point report of the SSADHD natural history study [28].

### Developmental, neuropsychologic, and clinical characteristics

All (100%) participants were born at term (after 37 weeks of gestation), weighed more than 2000 g at birth and did not endure any perinatal complications. None (0%) of the participants had microcephaly. The caregiver-reported symptom onset, which in most cases was an initial sign of developmental delay, occurred at a median (IQR) age of 0.5 (0.2–0.7) years (full range- 0–13 years). However, the median (IQR) age of diagnosis was 3.5 (2-5.4) years (full range 0.5–22.1 years). The majority of the study group currently have head control (98%), can sit unsupported (97%), and walk unsupported (90%). Subjects who reached these milestones were able to do so at median (IQR) ages of 6 (4–7), 11 (7–12), and 19 (16–30) months, respectively. Fifty-four (87%) study enrollees are currently able to express some words, and the median (IQR) age of their first expressed word was 28 (20–48) months (Table 1).

**Table 1:** Demographic, developmental, and clinical manifestations of individuals with SSADHD

**Characteristic**	SSADHD Study Group	Symptom Age of Onset
	**N = 62 (100%)**	Years (median, IQR)
**Sex**, M/F	29 (47%) / 33 (53%)	-
**Age**, median (IQR)	9.6 (5.4–14.5)	-
**Symptom onset (caregiver-reported)**	-	0.5 (0.2–0.7)
**Diagnosis**	-	3.5 (2.0-5.4)
**Developmental milestones achieved**		
Head control	61 (98%)	0.5 (0.3–0.6)
Sitting unsupported	60 (97%)	0.9 (0.6-1.0)
Walking unsupported	56 (90%)	1.6 (1.3–2.5)
First word	54 (87%)	2.3 (1.7-4.0)
**Neuropsychologic**		
FSIQ / DQ, mean ± SD (N = 32)	53.3 ± 13.6	-
Adaptive composite score, median (IQR) (N = 27)	65.0 (56.5–70.0)	-
Autism spectrum disorder diagnosis (N = 30)	17 (57%)	4.0 (2.0–12.0)
REEL-3 receptive age equivalent scores (N = 21)	28.0 (16.0-34.5)	-
REEL-3 expressive age equivalent scores (N = 21)	23.0 (9.7–33.5)	-
MABS percentile rank (N = 20)	13.0 (10.0-22.5)	-
**Epilepsy**	30 (48%)	9.0 (5.0–12.0)
Drug-resistant epilepsy	11 (18%)	-
EEG- diffuse background slowing (N = 55)	24 (44%)	-
EEG- epileptiform patterns (N = 55)	15 (27%)	
**Motor and movement**		
GMFCS 1–3/4–5	53 (85%) / 9 (15%)	-
Hypotonia	40 (64%)	0.25 (0.0-0.5)
Ataxia	25 (40%)	1.5 (1.0–2.0)
Dystonia/Dyskinesia	14 (22%)	1.1 (0.6–10.5)
**MRI (N = 39)**		
No cortical abnormality	28 (71%)	-
T2-weighted hyperintense signal- globus pallidi	18 (46%)	-
T2-weighted hyperintense signal- dentate nucleus	18 (46%)	-
**Psychiatric and Behavioral**		
CBCL/ABCL T-scores, mean ± SD (N = 27)	59.68 ± 8.13	-
OCD behaviors	22 (35%)	4.0 (2.0–9.0)
ADHD diagnosis	28 (45%)	5.0 (4.0-5.1)
Anxiety	25 (40%)	6.0 (3.0–13.0)
Psychosis	3 (5%)	5.0 (4.5–5.5)
**Sleep disturbances**		
Reported sleep disturbances	38 (61%)	1.0 (0.1–1.6)
CSHQ-defined sleep disturbances (N = 32)	25 (78%)	-
Total CSHQ score, mean ± SD (N = 32)	47.8 ± 8.1	-

ABCL- Adult Behavior Checklist; ADHD- Attention-deficit/hyperactivity disorder; CBCL- Child Behavior Checklist; CSHQ- Children’s Sleep Habits Questionnaire; DQ- Developmental quotient; EEG- Electroencephalography; FSIQ- Full scale intelligence quotient; GMFCS- Gross Motor Function Classification System; IQR- Interquartile range; MABS- Movement Assessment Battery for Children; MRI- Magnetic resonance imaging; OCD- Obsessive-compulsive disorder; REELS- Receptive expressive emergent language scales; SD- Standard deviation; SSADHD- Succinic semialdehyde dehydrogenase deficiency

The group’s mean ± SD full-scale intelligence quotient (FSIQ) is 53.3 ± 13.6. Composite adaptive scores are slightly higher, with a median (IQR) of 65 (56.5–70). Of the 30 subjects assessed by the ADOS-2, 17 (57%) were diagnosed with Autism Spectrum Disorders (ASD). The caregiver-reported median (IQR) age of autistic symptoms onset was 4 (2–12) years. According to the REEL-3 administered to 21 participants, the median (IQR) receptive language age equivalent scores [28 (16-34.5)] were significantly higher than the expressive [23 (9.7–33.5) (*p* < 0.001). The MABC-2 test, administered to 21 subjects, indicated they had a median (IQR) percentile rank of 15 (10.5–21). Notably, the raw score for each MABC-2 subtest [Manual dexterity (fine motor skills), Aiming and catching (gross motor skills), and Balance] is converted to a standardized score that is age-dependent. Scores of these subsets are added, resulting in a total score which is further converted to a standardized score and percentile rank based on normative data. A resultant score less than the 5th percentile indicates a “significant motor impairment” and that between the 6th to 15th percentile, a “risk of movement difficulty.” [36] These relatively good motor abilities were also reflected by the fact that 53 (85%) of the group were classified as I-III according to the Gross Motor Function Classification System (GMFCS) (Table 1).

**Fig. 4:**
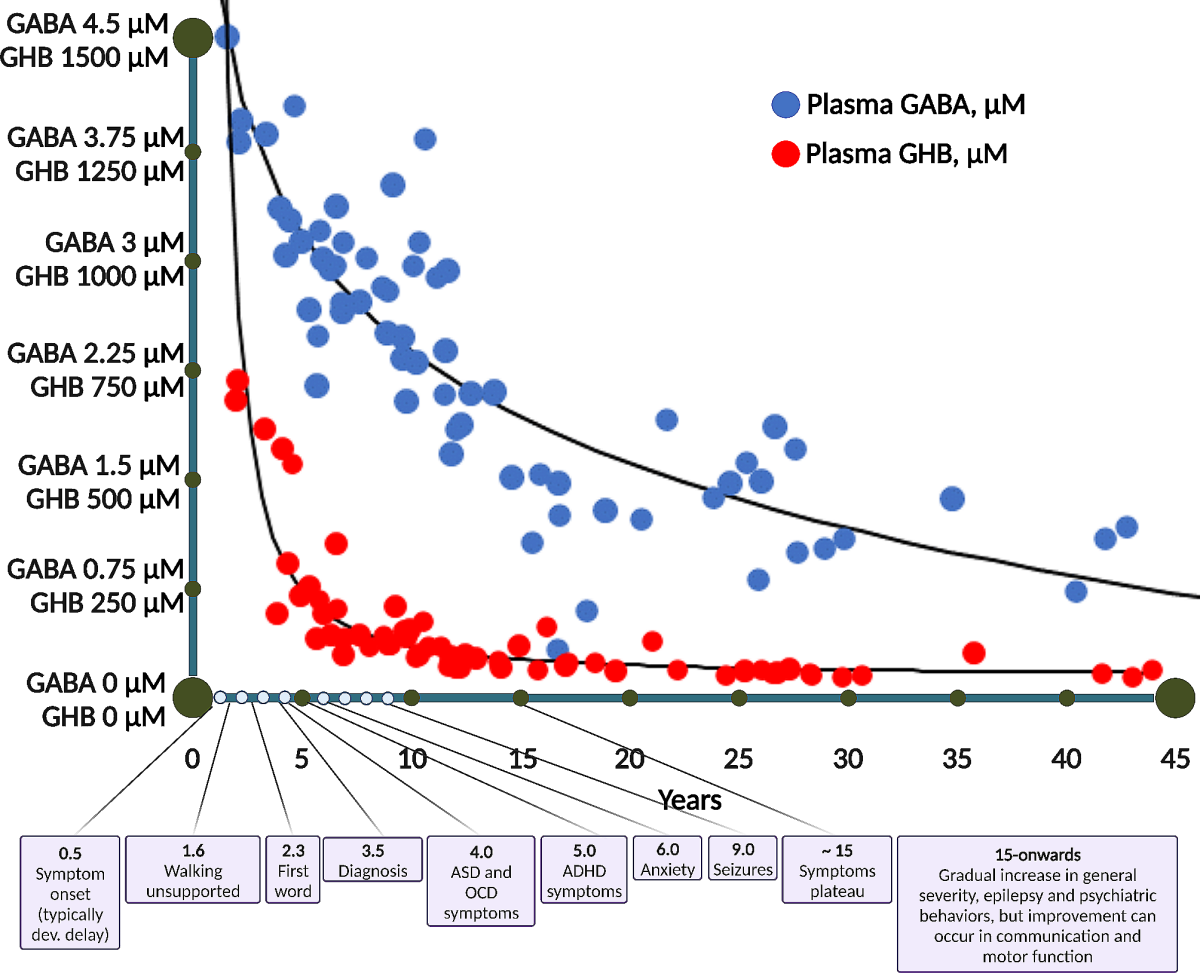
A schematic timeline of the median onset times of the principal clinical events in SSADHD alongside the decline in GABA and GHB. GABA- γ-aminobutyrate; GHB- γ-hydroxybutyrate

Thirty (48%) individuals from the study group had epilepsy, with seizure onset at a median (IQR) age of 9 (5–12) years. Of those with epilepsy, drug-resistance [61] to anti-seizure medications was noted in 11 (18%). Of those who underwent an EEG (*N* = 55), diffuse background slowing was seen in 24 (44%) and focal or multifocal epileptiform patterns in 15 (27%). Per neurological examination, 40 (64%) subjects from the group were determined to be hypotonic. Ataxia was identified in 25 (40%) individuals, and dystonia or dyskinesia in 14 (22%). Of the 39 participants who underwent an MRI, in 28 (71%) a cortical abnormality was not identified and in 18 (46%) T2-weighted hyperintense signals were seen in the globus pallidi and cerebellar dentate nuclei. Sleep disturbances were reported by SSADHD individuals or their caregivers during an in-person medical intake by 38 (61%) of the group, who reported they had onset at a median (IQR) age of 12 (1–19) months. The occurrence rate of sleep disturbances was even higher in the 32 subjects who completed the CHSQ questionnaire, as 25 (78%) of them were identified as having sleep-related pathology. Finally, as also reported by SSADHD subjects or their caregivers during the medical intake evaluation, descriptions of the study group’s psychiatric traits and their median (IQR) ages of onset consisted of 22 (35%) with OCD behaviors that were first seen at 4 (2–9) years, 28 (45%) with ADHD that was diagnosed at 5 (4-5.1) years, 25 (40%) with anxiety firstly noticed at 6 (3–13) years, and 3 (5%) with psychosis (hallucinations, delusions, ideas of reference, and social withdrawal) that presented at 5.5 (5-11.2) years (Table 1; Fig. 4).

### Correlation of clinical severity with biomarkers

The mean ± SD CSS of the study group was 17.2 ± 2.9, and that of the different CSS subscales was as follows: cognitive 3.3 ± 0.9, communication 2.6 ± 0.7, motor 3.7 ± 0.8, epilepsy 4.1 ± 1.2, and psychiatric 3.2 ± 1.2. The age of the study participants was significantly correlated with lower scores of the total CSS (*R*= -0.302, *p* = 0.03), and the epilepsy (*R*= -0.576, *p* < 0.001) and psychiatric (*R* = − 0.522, *p* < 0.001) domains, and higher scores in the communication (*R* = 0.310, *p* = 0.03) and motor (*R* = 0.368, *p* = 0.009) domains. The cognitive domain scores were not correlated with age (*R* = -0.054, *p* = 0.71). For this reason, we assessed the relationships between the CSS and the various genetic, metabolic, and neurophysiologic biomarkers after adjustment for age. The expression of *ALDH5A1* was significantly correlated with higher scores of the psychiatric CSS domain (*R* = 0.418, *p* = 0.05) but not with the other domains or the total CSS. All the remaining gene expression values of *ABAT*, *GLS*, *GLUL*, and *GLUD1* were not correlated with any CSS domain, apart from the sporadic inverse relation between ABAT and the cognitive domain (*R*= -0.568, *p* = 0.006) (Table 2). Plasma GABA was significantly correlated with the total CSS (*R* = 0.337, *p* = 0.02) and the CSS epilepsy (*R* = 0.402, *p* = 0.007) and psychiatric (*R* = 0.354, *p* = 0.01) domains. Almost similarly, higher plasma GHB values showed specificity for higher scores of the total CSS (*R* = 0.360, *p* = 0.05) and the psychiatric domain (*R* = 0.431, *p* = 0.01).

**Table 2:** Correlates of clinical severity with age, and age-adjusted biochemical, neurophysiologic, and neuroimaging biomarkers

Characteristic	Total CSS	Cognitive Domain	Communication Domain	Motor Domain	Epilepsy Domain	Psychiatric Domain
	R	R	R	R	R	R
	*p* value	*p* value	*p* value	*p* value	*p* value	*p* value
**Age**	-0.302	-0.054	0.31	0.368	-0.576	-0.522
	**0.03**	0.71	**0.03**	**0.009**	**< 0.001**	**< 0.001**
**Gene expression**, 2^ΔCT						
ALDH5A1 (N = 23)	0.154	0.038	-0.173	0.179	-0.113	0.418
	0.49	0.86	0.44	0.42	0.62	**0.05**
ABAT (N = 23)	-0.232	-0.568	0.272	0.156	-0.3	-0.089
	0.29	**0.006**	0.22	0.48	0.17	0.69
GLS (N = 23)	-0.008	-0.299	0.245	0.195	0.054	-0.1
	0.97	0.17	0.27	0.38	0.81	0.65
GLUL (N = 23)	-0.092	-0.104	-0.131	-0.325	-0.072	0.163
	0.68	0.64	0.56	0.14	0.75	0.68
GLUD1 (N = 23)	-0.064	-0.367	0.151	0.196	0.073	-0.137
	0.77	0.09	0.5	0.38	0.74	0.54
**Biochemical**						
Plasma GABA, µM/L (N = 43)	0.337	0.067	0.149	-0.149	0.402	0.354
	**0.02**	0.66	0.33	0.33	**0.007**	**0.01**
Plasma GHB, µM/L (N = 35)	0.36	0.145	0.18	-0.11	0.215	0.431
	**0.05**	0.44	0.34	0.95	0.25	**0.01**
**MRS**						
GABA/NAA, (BG, PCG, OCC)* (N = 16)	0.031	-0.225	0.201	-0.231	0.211	0.24
	0.9	0.37	0.42	0.35	0.4	0.33
**TMS**						
rMT, %MO (N = 23)	-0.312	-0.19	-0.262	-0.215	-0.095	-0.227
	0.15	0.39	0.23	0.33	0.67	0.31
CSP, ms (N = 21)	-0.085	-0.281	0.054	0.36	0.045	-0.234
	0.72	0.23	0.82	0.11	0.85	0.32
LICI, log (MEPt/MEPc) (N = 18)	-0.264	-0.295	-0.031	-0.211	-0.101	-0.123
	0.3	0.25	0.9	0.416	0.7	0.63

ABAT- 4-aminobutyrate aminotransferase; ADOS-2- Autism Diagnostic Observation Schedule-Second Edition; ALDH5A1- Aldehyde dehydrogenase 5 family member A1; ASD- Autism Spectrum Disorder; BG- basal ganglia; CSP- cortical silent period; EEG- Electroencephalogram; EEM- estimated marginal means adjusted for age; FSIQ- full-scale intellectual quotient; GABA- γ-aminobutyrate; GHB- γ-hydroxybutyrate; GLS- Glutaminase; GLUD1- Glutamate dehydrogenase 1; GLUL- Glutamate-ammonia ligase; IQR- Interquartile ratio; LICI- long interval intracortical inhibition; MEPc- motor evoked potential, condition; MEPt- motor evoked potential, test; MO- machine output; MRS- Magnetic resonance spectroscopy; ms- milliseconds; NAA- N-acetyl aspartate; OCC- occipital cortex; PCG- posterior cingulate gyrus; R- correlation coefficient; SD- Standard deviation; SE- standard error; SSADHD- succinic semialdehyde dehydrogenase deficiency; TMS- transcranial magnetic stimulation; Bold indicates significant. *Mean value of the GABA/NAA ratios measured from the basal ganglia, posterior cingulate gyrus, and occipital cortex)

Cerebral GABA, as measured by MRS-derived GABA/NAA ratio in the basal ganglia, posterior cingulate gyrus, and occipital cortex, and the TMS-derived metrics rMT, CSP, and LICI did not correlate with the total CSS or any of its domains.

### GHB analysis in mice

**Fig. 5:**
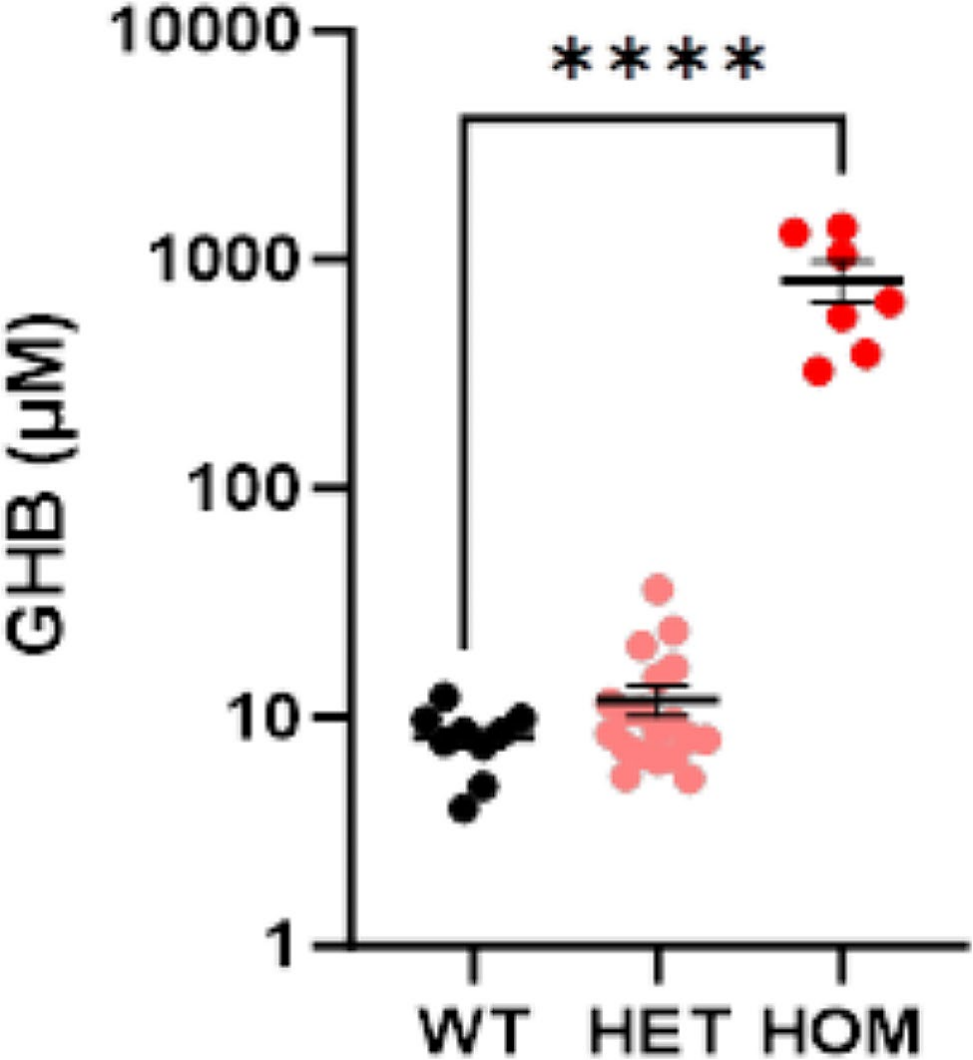
Quantification of whole blood GHB content in *aldh5a1*^*lox − STOP*^ mice. GHB analyses of whole blood obtained from wild-type (WT), heterozygous (HET), and homozygous mutant (HOM) *aldh5a1*^*lox − STOP*^ mice at postnatal age of 16 days. Quantification of GHB is expressed as µM. Individual data points, group mean, and SEM are shown. ****p < 0.0001, One-way ANOVA followed by Dunnett’s multiple comparisons test. Both male and female mice were used. *n* = 9 WT, 19 HET, 7 HOM mice

We found that HOM *aldh5a1*^*lox − STOP*^ mice have 812.7±165.3 µM GHB blood content, compared to 8.2±0.8 µM in WT littermates, an ∼ 100x increase in GHB blood level at this young age. Notably, HET mice GHB blood content was 11.86±1.78 µM, not significantly different from WT mice (Fig. 5). Overall, these results suggest that 50% SSADH (as in HET) is sufficient to regulate blood GHB content, but the total absence of SSADH (as in HOM) leads to significant blood GHB accumulation.

### SSADHD-derived human induced pluripotent stem cells (hiPSC)

Fibroblasts from three SSADHD patients and their sex-matched parental control (heterozygote and asymptomatic) were reprogrammed into iPSCs (Fig. 2a). We verified successful reprogramming by characterizing the expression of pluripotency markers: the iPSCs generated showed expression of pluripotency markers NANOG (Nanog homeobox x) and TRA-1-60 (podocalyxin), OCT4 (octamer binding transcription factor 4), and SOX2 (SRY-Box Transcription Factor 2) (Fig. 2b). Additionally, we differentiated the iPSCs into GABAergic neurons using induced DLX2 and ASCL1 and cultured these differentiating cells for 55 days. The neurons generated, which represent the first in vitro neuronal model of SSADHD, express the neuronal marker MAP2 (microtubule associated protein 2), as well as GABA (Fig. 2c) [56]. We found that induced GABAergic neurons showed the expected changes in GABA metabolism, with concomitant changes in gene expression, and that these could be reversed using CRISPR correction of the pathogenic variants or mRNA transfection. Furthermore, SSADH iPSCs were associated with excessive glutamatergic activity and corresponding synaptic excitation using microelectrode array recordings [56]. 

## Discussion

Stemming from an international effort, the 5-year natural history study of SSADHD has allowed us to deepen our knowledge of the genetic and metabolic pathophysiology and clinical presentation of this rare disorder. Quite remarkably, the 62 subjects enrolled in the study represent more than a quarter of the ∼ 200 SSADHD individuals described thus far [62, 63]. By reaching and exceeding this study’s primary objectives, we have also helped to set up further the platform for its secondary aims: development of targeted genetic therapies and inclusion as part of neonatal screening programs.

At this 5-year landmark of the study, we learned that caregiver-reported symptom onset was around six months of age, typically a developmental delay, but the diagnosis was not established until approximately 3.5 years of age, with several participants diagnosed in adolescence and adulthood. While the vast majority of the subjects were only mildly delayed in motor abilities, and currently, only 10% require support for walking, language delays are more prominent. From our bedside as well as longstanding clinical observations, expressive language deficits are more pronounced than receptive, and this was supported by the neuropsychiatric language assessments. Similar to what had been reported by a study dedicated to ASD in SSADHD [64], our results showed that from the 30 subjects assessed by ADOS-2, the gold standard for ASD diagnosis, 57% of the cohort was diagnosed with ASD. Interestingly, the caregiver-reported median (IQR) age of autistic symptoms onset in this population was 4 (2–12) years, which is higher than the age commonly reported in non-syndromic ASD, which is approximately 2.5 years [65]. With respect to epilepsy, our findings also corroborated a study focusing on epilepsy in SSADHD [66], showing that 48% of the group were diagnosed with epilepsy, which was defined as drug-resistant [61] in 18%. There have been reports of a relatively high Sudden Unexpected Death in Epilepsy (SUDEP) rate, mainly in adults with SSADHD [17]. Our cohort included one unfortunate case of SUDEP of a 14-year-old male.

As indicated by our findings, hypotonia is undoubtedly prevalent in SSADHD, especially in early childhood, but is not universal. Other movement disorders that were noted in up to a quarter of our group were an ataxic gait and, less commonly, dystonia and exertional dyskinesias. Sleep disorders of SSADHD individuals were reported by their caregivers in most cases. According to the CSHQ, a validated method for sleep disturbance assessment, an even higher occurrence rate (∼ 80%) of sleep disturbances existed in this population. Lastly, as reflected by neuropsychiatric intake and tests (CBCL/ABCL), the psychiatric traits that typified our group were hyperactivity and inattention, obsessive-compulsive behaviors, anxiety, and in a minority of cases, elements of psychosis such as delusions, hallucination, ideas of reference, and social withdrawal.

Considering the carrier rate for SSADHD is estimated at ∼ 1/340 [1] and the reported patients just above 200, there are potentially many existing undiagnosed SSADHD cases. This implies that there should be increased awareness to SSADHD in individuals presenting with any clinical manifestation listed above whose etiology is undetermined, especially if the onset age of that symptom coincides with that typical of SSADHD (Fig. 4).

A dysfunctional or truncated SSADH enzyme leads to the accumulation of GABA and GHB, as shown in humans [51] and our HOM *aldh5a1*^*lox − STOP*^ mice. Evidence also shows that as individuals with SSADHD age, their overall clinical severity worsens, as well as the severity of their epilepsy and psychiatric behaviors [21]. Moreover, it was demonstrated that GABA and other indices of cerebral inhibition decrease with age in SSADHD [67, 68]. This study’s biomarker-clinical severity relationship analyses showed that lower levels of age-adjusted plasma GABA and GHB significantly correlate with lower scores of the total CSS (reflecting an overall worst severity) and its psychiatric domain. In the case of GABA, this correlation was also noted with the epilepsy domain. It may be speculated that worse clinical outcomes in SSADHD may stem from greater disruption of cerebral excitation: inhibition homeostasis, as reflected by the decline in these inhibitory neurotransmitter levels. From a neurobiological standpoint, it has been shown that in SSADHD, the consistently high GABA and GHB levels promote the downregulation of their respective receptors [69–71]. A persistent hyperGABAergic environment may also negatively affect the cerebral GABA tone. According to recent evidence, GABA tonic currents play an essential role in cognitive functions, circadian rhythms, emotional regulation, sensory processing, and coordination [72]. A possible explanation of why we did not see specific relationships between clinical severity cerebral GABA or TMS-related metrics is the relatively lower number of subjects who underwent these tests. Notably, the non-specific relationship seen between clinical severity and expression of GABA-related genes expression could be linked to the autosomal recessive inheritance of this condition. Since SSADH is an oligomeric protein, the expression of *ALDH5A1* or other GABA-related genes and knowledge of the *ALDH5A1* variants is instructive only if the molecular effect on the SSADH protein is known. As shown in a study examining the genotype-to-protein-to-phenotype of SSADHD, worst severity was associated with lacking or a truncated SSADH (as opposed to having a SSADH composed of single homotetramers or multiple homo and heterotetramers), as well as having functional impairments in the enzyme’s catalytic functions (as opposed to stability, folding, or oligomerization) [22]. Nevertheless, plasma GABA and GHB may be used as biomarkers for prognostication and in clinical studies of SSADHD. It should be recognized that the significant biomarker-severity relationships found by our study are not absolute, as there were independent cases with opposing profiles, showing, for example, low values of plasma GABA and GHB but an overall mild clinical phenotype. Future studies utilizing patient-derived iPSCs and humanized mouse models can further help fill in the gaps in our understanding of the pathophysiology of SSADHD and enable mechanism-based therapeutic development.

## Conclusions

Endeavors of SSADHD natural history study, achieved by an international collaboration, have deepened our knowledge of the clinical phenotype and pathophysiology of this unique inherited metabolic disorder of GABA catabolism. Since GABA-related pathomechanism is involved in many other conditions such as epilepsy, ASD, and neurodegenerative disorders, findings from the natural history study also promote insight into their etiological processes. This serves as a powerful exemplar of combining clinical, translational, animal, molecular, and in silico studies surrounding a single severe neurometabolic disorder within the resources of the IDDRC.

## Data Availability

The datasets generated and/or analyzed during the current study are not publicly available due to privacy or ethical restrictions but are available from the corresponding author on reasonable request.

## Abbreviations

ABCL: Adult Behavior Checklist
ADOS-2: Autism Diagnostic Observation Scale-2
ASD: Autism Spectrum Disorders
BCH: Boston Children’s Hospital
CBCL: Child Behavior Checklist
CSHQ: Children’s Sleep Habits Questionnaire
CSP: Cortical silent period
CSS: Clinical severity score
EEG: Electroencephalography
EMG: Electromyography
GABA-γ: Aminobutyric acid
GBA-γ: Guanidinobutyrate
GHB-γ: Hydroxybutyrate
GMFCS: Gross Motor Function Classification System
HET: Heterozygous
hiPSC: Human induced pluripotent stem cells
HOM: Homozygous
iNTD: International Working Group on Neurotransmitter Related Disorders
IQR: Interquartile ranges
LICI: Long-interval cortical inhibition
MEG: Magnetoencephalography
MEP: Motor-evoked potential
MO: Machine output
MRI: Magnetic resonance imaging
MRS: Magnetic resonance spectroscopy
NAA: N-acetyl aspartate
NHS: Natural History Study
NIH: National Institutes of Health
REEL-3: Receptive Language and Expressive Language
rMT: The resting motor threshold
SEM: Standard error of the mean
SSADHD: Succinic semialdehyde dehydrogenase deficiency
SUDEP: Sudden Unexpected Death in Epilepsy
TMS: Transcranial magnetic stimulation
UCHH: University Children’s Hospital Heidelberg
UDB: Hospital Sant Joan Déu Barcelona Children’s Hospital
WT: Wild-type
